# Mobile Health Apps That Act as Surgical Preparatory Guides: App Store Search and Quality Evaluation

**DOI:** 10.2196/27037

**Published:** 2021-11-30

**Authors:** Naga Sindhura Gadde, Kevin Yi-Lwern Yap

**Affiliations:** 1 Department of Public Health School of Psychology and Public Health La Trobe University Melbourne Australia; 2 Department of Pharmacy Singapore General Hospital Singapore Singapore

**Keywords:** mHealth apps, surgical apps, surgery preparation, operating room personnel, quality assessment, quality evaluation, perioperative, operative, mobile health, surgery, post-operative

## Abstract

**Background:**

Mobile health (mHealth) apps are becoming increasingly common in surgical practices for training, education, and communication. Factors leading to increased delays, morbidity, and mortality in surgery include inadequate preoperative patient preparation due to a failure to identify patients and procedure details, and missing instruments and equipment required for the procedure. Many apps are available for supporting preoperative, intraoperative, and postoperative care. However, there is a lack of studies that assess the quality of apps that act as surgical preparatory guides.

**Objective:**

The aim of this study is to evaluate the quality of apps that act as surgical preparatory guides for operating room personnel through an in-house quality assessment tool.

**Methods:**

The quality assessment tool comprises 35 questions categorized into 5 sections: (1) engagement (customization, interactivity, target audience; 19 points), (2) functionality (performance, ease of use, navigation; 12 points), (3) aesthetics (layout, visual appeal; 6 points), (4) information (quality and quantity of information, visual information, credibility; 29 points), and (5) privacy and security (4 points). An app search was conducted in the Australian Apple and Google Play stores using the following keywords: “surgical apps”, “surgical preferences”, “surgeon preferences”, “operating room”, and “perioperative procedures”. The overall total scores and scores for each section were reported as medians and IQRs, expressed as raw scores and percentages.

**Results:**

A total of 5 unique apps were evaluated on both iOS and Android platforms. The median overall score across all apps was 35/70 (50%; IQR 38.6%-64.3%). ScrubUp (48/70, 69%) and MySurgeon (42/70, 60%) had the highest overall scores, followed by PrefCard (35/70, 50%) and Scrubnote (28/70, 40%). The lowest scoring app was BrainPadd (26/70, 37%). The sections with the highest median scores, in decreasing order, were privacy and security (4/4, 100%; IQR 75%-100%), aesthetics (5/6, 83%; IQR 75%-91.7%), engagement (15/19, 79%; IQR 57.9%-86.8%), functionality (7/12, 58%; IQR 29.2%-75%), and information (5/29, 17%; IQR 15.5%-34.5%). Most apps scored well (4/4, 100%) on privacy and security, except for Scrubnote (2/4, 50%). ScrubUp received a perfect score for aesthetics (6/6, 100%). MySurgeon (17/19, 90%) had the highest engagement score, while ScrubUp and MySurgeon had the highest functionality scores (9/12, 75% each). All apps scored below 50% for the information section, with ScrubUp having the highest score of 13/29 (45%).

**Conclusions:**

ScrubUp and MySurgeon had the highest quality scores and can be used as adjuncts to hospital protocols by operating room personnel for their surgical preparation. Developers are encouraged to develop appropriate apps for surgical preparation based on relevant guidelines and standards, as well as the quality evaluation criteria in our tool. Operating room personnel can also use this tool as a guide to select and assess their preferred apps in their practices.

## Introduction

As the global digital health market continues to flourish, technological innovations such as electronic medical record systems, laboratory and clinical information systems, mobile apps, health information technology, wearable devices, telehealth, and telemedicine are becoming more common in the health care setting to improve health service delivery and quality [1]. Mobile health (mHealth) technologies such as mobile phones and patient monitoring devices are becoming increasingly common in medical and surgical practices for training, education, and communication [1,2]. Surgical processes are complex, and a variety of factors may result in surgical delays and cancellations [3]. Major factors leading to increased delays, morbidity, and mortality in surgery include inadequate preoperative patient preparation resulting from a failure to identify patients and procedure details, and missing instruments and equipment required for the procedure [4]. Similarly, perioperative nurses face pressures to balance between maintaining operating room schedules and the surgeon’s demands due to time restrictions and the potential to underestimate the time required to operate [5]. Furthermore, newly graduated nurses who are uncertain about the intensified and unfamiliar situations during the initial stages of their clinical practice may face additional pressures in addressing emerging and complex technology, resulting in technological stress and surgical errors [6]; these pressures are in addition to trying to cope with the demands of new clinical placements and advancing their professional careers at the same time [7,8].

Surgical preparation tools such as preassessment clinics and the World Health Organization Surgical Safety Checklist aim to minimize adverse events and errors in the operating room [3,9]. However, in some developing countries, surgical teams are still unable to use the safety checklists effectively [10]. With an increasing global trend of smartphone users to a predicted 3.8 billion users in 2021 [11], it is envisaged that smartphone apps may be useful to surgical teams, newly graduated health care professionals, or health care trainees to improve their awareness of and attitudes toward surgical safety practices since these apps can be accessed anytime and anywhere [10,12]. Some features of such apps that may enhance the usability of these safety checklists include the ability to customize surgical preparatory notes according to user preferences, provide comprehensive step-by-step information on preoperative and postoperative procedures, and provide clear and accurate visual explanations of the surgical procedures, for example, through images or videos. Furthermore, smartphone apps can act as quick reference guides that provide various clinical resources as a part of training and education for health care trainees, students, residents, fellows, and surgeons during their clinical practices to ultimately improve communication, system efficiency, and patient safety [1,13-15]. Studies have shown that many apps are available for supporting preoperative, intraoperative, and postoperative care [16], and that health care professionals working in surgical care and app developers are also producing innovative apps to aid surgical teams in education, training, and practice [15].

Despite the widespread availability of mHealth apps, the literature has focused mostly on the prevalence and evaluation of communication, education, clinical, and diagnostic apps for physicians; health and medication monitoring apps for patients; and healthy living apps for diet, exercise, pregnancy, and heart rate monitoring for laypersons [13]. In the surgical domain, some studies have focused on the use of mobile patient health record apps and apps for diagnosis in the perioperative setting (eg, smartphone-based electrocardiograms, pulse oximetry, and blood glucose monitoring), while others have concentrated on the use of medical reference and perioperative crisis event management apps to improve care quality and safety in patient care, as well as on apps that facilitate patient monitoring and follow-up in the postoperative period [17]. The use of smartphones to promote better communication (eg, text messaging, emails) among the care team has also been studied [17]. However, there is a lack of studies that assess the quality of apps that act as surgical preparatory guides. Surgical preparatory guides help to support the surgical team in preparing the operating room and the patients before each procedure. They can consist of checklists of tools and equipment (eg, dressings, drapes, disposables, surgical instruments), information on preoperative safety procedures (eg, checking consent forms and diagnostic images, identification of patients and surgical sites), information about surgical preparatory steps (eg, putting up drapes, sterilizing surgical sites, preparing the patient and operating room, correct positioning of the patient), information on postoperative steps after each procedure (eg, recording correct counts of instruments, procedure names, specimens collected, equipment issues), and related reference sources, among others. Furthermore, there are concerns that there is no control on the quality of the apps, nor is there any regulated body that oversees the validity of the content unless the app is considered a medical device [18]. On the other hand, the lack of proper information on the quality of such apps and their content makes it difficult for users to identify the most useful apps, and there is also a risk that users may access misguided or misleading information [12,19]. Therefore, the objective of this study is to develop a quality assessment tool to evaluate apps that act as surgical preparatory guides for operating room personnel (nurses, surgical technicians, circulating nurses and technicians). The aim is to provide a recommendation of apps that may be useful to operating room personnel during their training and initial stages of clinical practice to improve patient safety and health communication within the surgical team.

## Methods

### Development of the Quality Evaluation Tool

The overall framework of the quality evaluation tool was adapted from the Mobile Application Rating Scale (MARS) [20], with modifications made to some criteria to fit the evaluation of apps for surgical preparation based on relevant articles found from a keyword search in PubMed (“surgical apps” OR “surgical applications” OR “ surgical safety” AND “quality tools” OR “quality scale” OR “assessment criteria” OR “evaluation”) [15,16,18,21-24]. In addition, we used relevant articles that reported quality assessment tools or criteria for evaluating general mHealth apps to refine the quality evaluation criteria of our tool [25-43].

The tool was comprised of 35 questions categorized into 5 sections: (1) engagement (customization, interactivity, target audience; 19 points), (2) functionality (performance, ease of use, navigation; 12 points), (3) aesthetics (layout, visual appeal; 6 points), (4) information (quality and quantity of information, visual information, credibility; 29 points), and (5) privacy and security (security, privacy; 4 points) (Multimedia Appendix 1). The maximum possible score was 70 points.

### Selection of Apps

**Figure 1:**
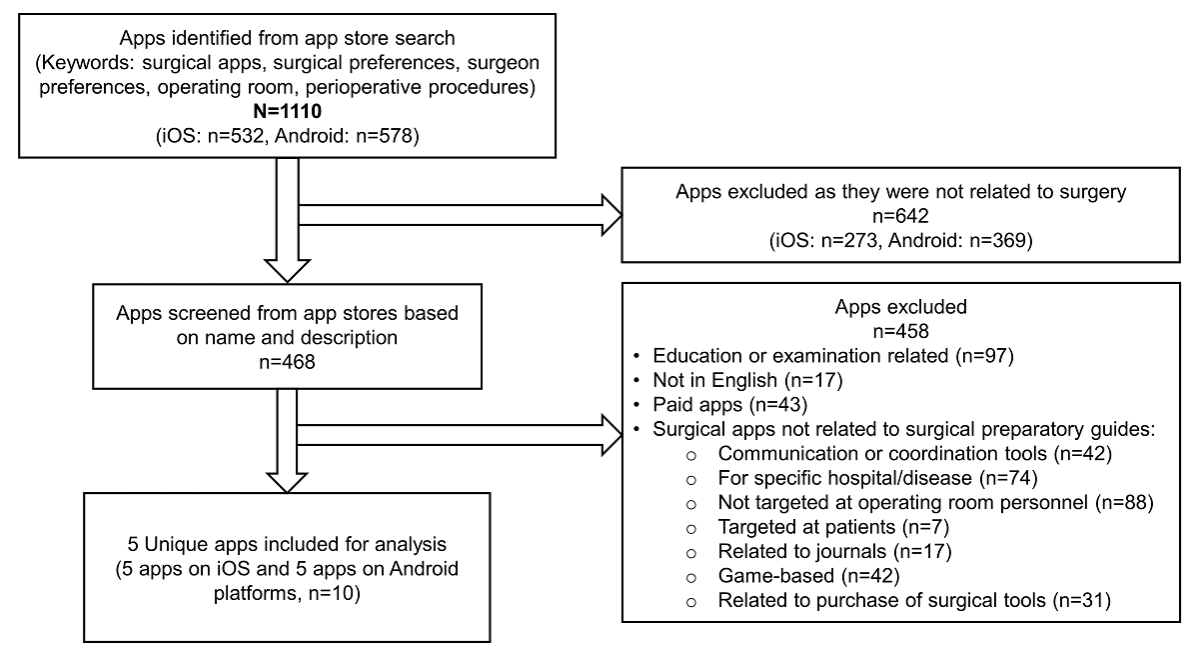
Flowchart summary of the app screening process.

We conducted an app search in August 2020 on the Australian Apple (iOS) and Android (Google Play) app stores with the keywords “surgical apps”, “surgical preferences”, “surgeon preferences”, “operating room”, and “perioperative procedures”, which resulted in an identification of 1110 apps (Figure 1). Among these apps, a total of 642 apps were not related to surgery and were excluded from screening. Among the 468 apps that were screened based on app name and description, a total of 458 apps were excluded based on the following exclusion criteria: education or examination-related, language other than English, and requiring a subscription or payment for access. Surgical-related apps that were not related to surgical preparatory guides were also excluded including communication or coordination tools, apps specific to a hospital or disease, apps targeting non–operating room personnel or patients, game-based apps, journal-related apps, and apps advocating for the purchase of surgical tools. There were 5 unique surgical apps that were evaluated on both iOS and Android platforms. The iOS and Android versions of the apps were evaluated on an iPhone 7 (Apple Inc) and Oppo A37F and X9079 (Guangdong Oppo Mobile Telecommunications Corp., Ltd) phones, respectively.

### Evaluation and Data Analysis

The apps were evaluated independently by 5 individual raters. Any discrepancies in scores were resolved by a discussion among all the raters, and the final scores were used for analysis. The iOS and Android versions for each unique app were not treated differently; the results were not reported in relation to the different platforms, but per unique app instead. We computed descriptive statistics for the overall total scores and the scores for each section of the apps using SPSS, version 26 (IBM Corporation) and reported these scores as medians and IQRs, expressed as the raw scores and percentages.

## Results

The median overall score across all the apps was 35/70 (50%; IQR 38.6%-64.3%). The apps that had the highest overall scores were ScrubUp (48/70, 69%) and MySurgeon (42/70, 60%), while the lowest scoring app was BrainPadd (26/70, 37%). Among all the apps, only 3 (ScrubUp, MySurgeon, and PrefCard) had an evaluation score of 50% or above (Table 1). The sections with the highest scores, in decreasing order, were privacy and security (4/4, 100%), aesthetics (5/6, 83%), engagement (15/19, 79%), functionality (7/12, 58%), and information (5/29, 17%).

In general, 4 apps took into consideration privacy and security aspects of their features, achieving the maximum score of 4/4 (100%). Only Scrubnote scored 2/4 (50%) as it did not explicitly state any privacy policy. All apps had a security login function for user authentication purposes.

**Table 1:** Evaluation scores of the apps.

		Evaluation scores for the surgical preparation apps, ranked from first (left) to last (right), points (%)						Scores across all the apps	
		ScrubUp^a^	MySurgeon^b^	PrefCard^c^	Scrubnote^d^	BrainPadd^e^	Median (%)		IQR (%)
**Engagement (out of 19)**		16 (84)	17 (90)	15 (79)	14 (74)	8 (42)	15 (79)		11-16.5 (57.9-86.8)
	Customization (out of 14)	12 (86)	13 (93)	11 (79)	10 (71)	4 (29)	11 (79)		7-12.5 (50-89.3)
	Interactivity (out of 4)	3 (75)	3 (75)	3 (75)	3 (75)	3 (75)	3 (75)		3-3 (75-75)
	Target audience (out of 1)	1 (100)	1 (100)	1 (100)	1 (100)	1 (100)	1 (100)		1-1 (100-100)
**Functionality (out of 12)**		9 (75)	9 (75)	7 (58)	3 (25)	4 (33)	7 (58)		3.5-9 (29.2-75)
	Performance (out of 2)	1 (50)	2 (100)	2 (100)	1 (50)	1 (50)	1 (50)		1-2 (50-100)
	Ease of use (out of 8)	6 (75)	6 (75)	3 (38)	1 (13)	1 (13)	3 (38)		1-6 (12.5-75)
	Navigation (out of 2)	2 (100)	1 (50)	2 (100)	1 (50)	2 (100)	2 (100)		1-2 (50-100)
**Aesthetics (out of 6)**		6 (100)	5 (83)	5 (83)	4 (67)	5 (83)	5 (83)		4.5-5.5 (75-91.7)
	Layout (out of 4)	4 (100)	3 (75)	3 (75)	3 (75)	3 (75)	3 (75)		3-3 (75-75)
	Visual appeal (out of 2)	2 (100)	2 (100)	2 (100)	1 (50)	2 (100)	2 (100)		1.5-2 (75-100)
**Information (out of 29)**		13 (45)	7 (24)	4 (14)	5 (17)	5 (17)	5 (17)		4.5-10 (15.5-34.5)
	Quality and quantity of information (out of 12)	4 (33)	2 (17)	1 (8)	2 (17)	1 (8)	2 (17)		1-3 (8.3-25.0)
	Visual information (out of 9)	4 (44)	0 (0)	0 (0)	0 (0)	0 (0)	0 (0)		0-2 (0-22.2)
	Credibility (out of 8)	5 (63)	5 (63)	3 (38)	3 (38)	4 (50)	4 (50)		3-5 (37.5-62.5)
**Privacy and security (out of 4)**		4 (100)	4 (100)	4 (100)	2 (50)	4 (100)	4 (100)		3-4 (75 – 100)
	Privacy (out of 2)	2 (100)	2 (100)	2 (100)	0 (0)	2 (100)	2 (100)		1-2 (50-100)
	Security (out of 2)	2 (100)	2 (100)	2 (100)	2 (100)	2 (100)	2 (100)		2-2 (100-100)
Total score (out of 70)		48 (69)	42 (60)	35 (50)	28 (40)	26 (37)	35 (50)		27-45 (38.6-64.3)

^a^Allis Technology Pty Ltd.

^b^Mederi Services, LLC.

^c^Headjam Pty Ltd.

^d^Scrubnote LLC.

^e^Connexxus LLC.

Overall, all apps scored well in the aesthetics section (median 5/6, 83%; IQR 75%-91.7%). Only 1 app (ScrubUp) received a perfect score (6/6, 100%), while another app (Scrubnote) scored the lowest (4/6, 67%). In general, the majority of the apps scored well for visual appeal, with consistent colors and fonts, and a clear organization of the content on the screen interface. However, there were some minor difficulties in locating and selecting some icons on most apps, except ScrubUp; thus, these apps only scored 3/4 (75%) in terms of layout.

The median engagement score for the apps was 15/19 (79%; IQR 57.9%-86.8%). MySurgeon (17/19, 90%) had the highest engagement score, followed by ScrubUp (16/19, 84%). BrainPadd scored the lowest in engagement (8/19, 42%). All the apps scored 3/4 (75%) for interactivity as they only allowed one method of feedback about the app. All of the content in the apps was appropriate for their target audiences (1/1, 100%). In terms of customization (median score 11/14, 79%), all apps allowed users to store personalized notes according to their own preferences and the preferences of their surgical team members. BrainPadd was the only app that did not allow users to edit any preloaded information about surgical preparatory procedures or tools, nor add any additional details or images to the preloaded information, thus scoring the lowest (4/14, 29%). There were only 2 apps that allowed limited customization of notifications (MySurgeon, PrefCard) and syncing of scheduled reminders or alerts (MySurgeon, ScrubUp).

Functionality was the second lowest scoring section, with a median score of 7/12 (58%; IQR 29.2%-75%). While more than half of the apps (ScrubUp, PrefCard, BrainPadd) scored well due to their consistency in navigation (2/2, 100%), only 2 apps (MySurgeon, PrefCard) did not have any technical issues (2/2, 100% each). The top scoring apps in this section (ScrubUp and MySurgeon, 9/12, 75% each) also scored the highest for ease of use (6/8, 75% each). These apps enabled users to access saved information without the need for internet access and also provided useful help sections for navigating the apps. By contrast, Scrubnote (3/12, 25%) and BrainPadd (4/12, 33%) scored the lowest for this section.

The information section was the lowest scoring section, with a median score of 5/29 (17%; IQR 15.5%-34.5%). All the apps scored below 50% in this section, with the highest scoring app (ScrubUp) having a score of only 13/29 (45%). PrefCard scored the lowest in this section (4/29, 14%). None of the apps had a preoperative surgical safety checklist or a checklist of postoperative steps to be completed after the surgical procedure. ScrubUp was the only app that had preloaded images of the surgical instruments/tools displayed to users, resulting in a score of 4/9 (44%) in terms of visual information. With regard to credibility, ScrubUp and MySurgeon had the highest scores (5/8, 63%), even though none of the apps provided any information on funding.

## Discussion

### Principal Findings

This study analyzed 5 apps that act as surgical preparatory guides for operating room personnel who might need them in their practices, such as those in training or new to the operating room. Among them, 4 apps (ScrubUp, MySurgeon, Scrubnote, PrefCard) could be used by multiple surgical specialties, while BrainPadd was specific to plastic surgery. As surgery procedures are becoming more advanced and complex, the operating room environment needs to be coordinated efficiently through effective communication among the surgical team members [44]. Therefore, the evaluation criteria in the customization section of our developed tool were intended to support the communication of surgical team members in the operating room. Our tool also evaluated the engagement of the apps in terms of customization and interactivity, as well as the user-friendliness of the apps. In general, the engagement scores of all the apps were relatively high. All the apps allowed users to store personalized notes. Users were also able to provide feedback about the apps through at least one form of contact to the company or developer (eg, contact number, email, feedback form). In terms of user-friendliness, the top scoring apps, ScrubUp and MySurgeon, provided useful help sections for users to navigate the apps. Therefore, these apps could potentially be used by the surgical team with little extra training or resources. On the other hand, developers of the other evaluated apps could improve the functionality features, such as providing more detailed instructions or user guides on how to use the apps and providing more obvious navigation links between screens. Furthermore, the importance of checklists was highlighted in several studies as an effective communication tool that could impact operating room efficiency and reduce delays and errors in surgical settings [45-47]. The Surgical Safety Checklist by the World Health Organization was developed to address this challenge of minimizing common and avoidable risks in the operating room before, during, and after the surgery process [9]. Thus, the need for a surgical safety checklist was also evaluated as part of the information section when evaluating the surgical preparatory apps in this study.

Providing evidence-based information is one of the important criteria for medical apps, and this can be said for surgical preparatory apps as well. Studies have shown that health care professionals are more inclined to use apps that can provide current and up-to-date information at the point of care in clinical practice [13]. Similarly, other studies have also reported that users value apps that can provide them with immediate access to information [48]. Our evaluation tool attempted to address these factors in the information section by assessing the up-to-dateness of the app content, as well as the presence of preloaded information and evidence-based references. Unfortunately, none of the apps evaluated in this study contained preoperative surgical safety or postoperative procedural checklists, nor did they provide references for their information. Even though ScrubUp scored the highest in the information section, it only contained checklists of the surgical instruments and tools needed and preloaded images. Interestingly, none of the apps had included any preloaded or linked videos to explain the surgical preparatory procedure. Video-based learning is a useful and effective way of learning about surgical preparation, especially among residents [49]. Although the quality of surgical videos on video sharing sites such as YouTube can be improved, this form of learning presents an opportunity and can be considered for inclusion by app developers, if the videos are accurate, reliable, and evidence-based [50].

In our study, PrefCard was ranked third based on its overall evaluation score. We observed that certain features of the app could only be accessed by users who were on the app’s list of affiliated hospitals. As the raters in our study were not from the list of affiliated hospitals, app features such as customization, functionality, and quantity and quality of the content were evaluated based on the images and descriptions provided in the app stores and the developer website. Other criteria related to functionality, such as performance of the app (eg, technical bugs or crashes), having an autocomplete feature, and accessing saved information in offline mode might have been scored differently from another user who had full access to all the features of the app. Furthermore, during the evaluation of BrainPadd, there were some technical issues with regard to downloading and accessing the app at the later stages; hence, the raters evaluated some criteria based on the description on its website. As with PrefCard, users who have full access to BrainPadd might also score the app differently.

### Limitations

A limitation of this study was that it only evaluated apps available in the English language and from the Australian app stores. Therefore, our results would need to be extrapolated with caution when applied to apps in other countries. In addition, this study only evaluated the app features that were free. There were some features in the apps that were available as in-app purchases, and these were not included in our evaluation. The evaluation criteria in our tool were developed specifically to assess apps that were meant to be surgical preparatory guides, and not all surgical apps as the variety of surgical apps was too broad. Thus, evaluators who want to use this tool to conduct their own evaluations of surgical apps would have to modify or adapt the criteria to fit their scope of evaluation. Moreover, usability and user acceptance studies or trials are beyond the scope of this study due to time limitations. Future studies should include evaluating the receptivity and acceptance of these apps among potential users, as well as involving appropriate health care professionals as evaluators, such as those in the surgical team. Lastly, this study did not take into account any updates to the apps after their evaluation in August 2020. Any updated versions of the apps might lead to different scores for the individual sections and the overall quality scores. Users should consider any new or updated features of the apps when interpreting our results.

### Conclusion

This study, we developed a tool for evaluating apps that act as surgical preparatory guides. Based on our evaluation, ScrubUp and MySurgeon are among the apps with better scoring features and can be used as adjuncts to existing hospital protocols for surgery preparation. In addition, the evaluation criteria in our tool can provide a form of guidance for operating room personnel, surgical professionals, and trainees to evaluate their preferred apps in the future. Similarly, app developers are encouraged to develop apps that follow relevant guidelines and standards, as well as the quality criteria in this tool, so that better quality apps that are reliable and incorporate evidence-based content can be used for surgical practices. Where appropriate, we also encourage app developers to submit their apps to relevant regulatory agencies for further evaluation and feedback.

## Appendix

Multimedia Appendix 1 Quality evaluation tool developed for surgical preparation apps.

## Abbreviations

MARS: Mobile App Rating Scale
mHealth: mobile health
