# Supplementary material for: Mobile Health Apps That Act as Surgical Preparatory Guides: App Store Search and Quality Evaluation
Source: JMIR Perioper Med. 2021 Nov 30;4(2):e27037. doi: 10.2196/27037 (PMC8672284; doi:10.2196/27037)
Supplement: Multimedia Appendix 1 [file periop_v4i2e27037_app1.docx]

**Appendix A.** Quality evaluation tool developed for surgical preparatory apps.

| **QUALITY EVALUATION TOOL FOR SURGICAL PREPARATION APPS** | |
| --- | --- |
| **TOTAL SCORE : /70** | |
| ***SECTION 1: ENGAGEMENT: SCORE= /19 POINTS*** | |
| 1. ***Customisation: Max score (14 points)*** | |
| 1 | Does the app allow users to customise their notification features?  (2) Allows comprehensive customisation (1) Allows limited customisation (0) Does not allow any customisation |
| 2 | Does the app allow users to input/store personalised information library/notes according to their **own** preferences (e.g. surgical preparatory notes, photos etc)?  (2) Allows input/storage of personalised information library/notes according to their own preferences (1) Allows input/storage of personalised information library/notes but only to a limited extent (0) Does not allow input/storage of personalised information library/notes |
| 3 | Does the app allow users to input/store personalised information library/notes according to their **surgical team members**’ preferences (e.g. surgical preparatory notes, photos etc)?  (2) Allows input/storage of personalised information library/notes according to their surgical team members’ preferences (1) Allows input/storage of personalised information library/notes according to surgical team members’ preferences, but only to a limited extent (0) Does not allow input/storage of personalised information library/notes |
| 4 | Does the app allow users to edit pre-loaded information about **surgical preparatory procedures/steps** (e.g. preparing the patient, theatre, positioning of the patient)?  (2) Allows edit of surgical preparatory procedural information  (1) Does not allow edit of the pre-loaded information (0) There is no pre-loaded information |
| 5 | Does the app allow users to edit pre-loaded information about **surgical preparatory tools** (e.g. equipment/instrument)?  (2) Allows edit of information about surgical preparatory tools (1) Does not allow edit of the pre-loaded information (0) There is no pre-loaded information |
| 6 | Does the app allow users to add **additional information** to the pre-loaded surgical preparatory procedures and tools?  (1) Allows the adding of additional information to the pre-loaded procedures and tools (0) Does not allow the adding of any additional information |
| 7 | Does the app allow users to add the **additional images** to the pre-loaded surgical preparatory procedures and tools?  (1) Allows to add additional images to the pre-loaded procedures and tools (0) Does not allow to add any additional images |
| 8 | Does the app allow users to schedule **reminders/alerts** related to the surgical procedures (e.g. synced with external calendar, integrated with SMS)?  (2) Advanced reminders can be set (synced with external calendar/ integrated with SMS) (1) Basic reminders can be set (e.g. locally on device) (0) Reminders cannot be set |
| ***B) Interactivity: Max score (4 points)*** | |
| 9 | Does the app allow users to provide feedback (e.g. contact number, email, contact forms, live chats)?  (2) Allows provision of feedback through more than one option (1) Allows provision of feedback through only one option (0) Does not allow provision of feedback |
| 10 | Does the app allow sharing of surgical information among users (e.g. e-mails, SMS, in app contacts)?  (2) Allows sharing via more than one option (1) Allows sharing via only one option (0) There is no sharing option available |
| ***C) Target audience: Max score (1 point)*** | |
| 11 | Is the app content appropriate for its stated target audience?  (1) The content is appropriate  (0) The content is inappropriate |
| ***SECTION 2: FUNCTIONALITY: SCORE= /12 POINTS*** | |
| 1. ***Performance: Max score (2 points)*** | |
| 12 | Does the app features (functions) and components (buttons/menus) work well without any technical issues (e.g. bugs, crashes, hang issues, device support issues, etc)?  (2) Works well with no technical issues (1) Works relatively well with few technical issues (0) Does not work properly with many technical issues |
| 1. ***Ease of use: Max score (8 points)*** | |
| 13 | Is the app intuitive and easy to use?  (2) Simple and intuitive to use  (1) Requires some time/effort in learning how to use  (0) Complicated to use with limited instructions |
| 14 | Are users able to access saved information when internet is not available (i.e. offline mode)?  (2) All saved information is accessible in offline mode  (1) Limited information is accessible in offline mode (0) No information is accessible in offline mode |
| 15 | Does the app have an autocomplete feature (e.g. suggestions based on inputs of procedures, equipment and tools, etc)?  (2) App has autocomplete feature for all the information (1) App has autocomplete feature to limited information (0) Autocomplete feature is absent |
| 16 | Does the app provide a help section or user guide/instructions?  (2) The app provides a help section or user guide that is presented in a simple and clear manner (1) The app provides a help section or user guide but is presented in a complex and unclear manner (0) The app does not provide a help section or user guide |
| ***C) Navigation: Max score (2 points)*** | |
| 17 | Is navigating between screens consistent with all the necessary links present?  (2) Consistent with obvious links throughout (1) Consistent but with few missing links (0) Inconsistent with many links missing |
| ***SECTION 3: AESTHETICS: SCORE= / 6 POINTS*** | |
| 1. ***Layout:*** ***Max Score (4 points)*** | |
| 18 | Is arrangement of the buttons/icons/menus simple and clear?  (2) Simple and clear  (1) Satisfactory with minor problems in selecting and locating buttons/icons/menus (0) Difficult to locate and select the buttons/icons/menus |
| 19 | Is the arrangement of the content/information on the screen clearly organised?  (2) Clearly organised (1) Some sections are not as organised as others  (0) Very disorganised |
| 1. ***Visual appeal: Max Score (2 points)*** | |
| 20 | Does the app user interface look visually appealing with consistent themes (e.g. fonts and colours)?  (2) Visually appealing with consistent themes (1) Appealing with minor inconsistencies in themes (0) Not visually appealing and poorly designed with obvious differences in themes |
| ***SECTION 4: INFORMATION: SCORE= /29 POINTS*** | |
| 1. ***Quality and quantity of information: Max score (12 points)*** | |
| 21 | Is the app content updated regularly and have a recent updated date?  (2) Updated regularly and states its last updated date (1) Only states how regularly it is updated, without any specific date or provides only a specific date without stating frequency of updates (0) There is no update information stated |
| 22 | Does the app have comprehensive pre-loaded step-by-step information on pre-operative procedures such as preparation/positioning of the patient for each procedure (e.g. putting drapes, sterilising the surgical sites, correct patient positioning, etc)?  (2) Comprehensive pre-loaded information is provided (1) Pre-loaded information is provided but not comprehensive (0) No pre-loaded information available |
| 23 | Does the app have a pre-operative surgical safety checklist (e.g. checking consent forms, diagnostic images, identification of patients, surgical sites, procedures, blood types, equipment issues, etc)?  (2) Pre-operative safety checklist is present for all the procedures (1) Pre-operative safety checklist is present only for a few procedures (less than half) (0) Pre-operative safety checklist is absent |
| 24 | Does the app have a checklist of tools/equipment required for prepping the patient/operating room for each procedure (e.g. dressings; drapes; disposables; instruments such as forceps, retractors, scissors, needle holders; equipment such as light source, monitors, suction units, specimen bags; others such as resuscitation trolleys, anaesthetic gases, sutures and needle holders, solutions, etc)?  (2) Checklist of tools/equipment for each procedure is present (1) Checklist of tools/equipment is present only for a few procedures (less than half) (0) Checklist of tools/equipment is absent |
| 25 | Does the app have a checklist of post-operative steps to be completed immediately after each procedure and before the patient leaves the operating room (e.g. recording correct count of instruments, needles, sponges, procedure names, specimen collected, equipment problems, etc)?  (2) Checklist of post-operative steps is present for each procedure (1) Checklist of post-operative steps is present only for a few procedures (less than half) (0) Checklist of post-operative steps is absent |
| 26 | Does the app provide sources/references for the information related to surgical preparatory procedures (e.g. surgical preparatory procedural steps, instruments, equipment, etc)?  (2) The app provides sources/references for all surgical preparatory information (1) The app provides sources/references to only some surgical preparatory information (0) The app does not provide any sources/references |
| 1. ***Visual information: Max score (9 points)*** | |
| 27 | Does the app contain **clear** visual explanations for each surgical procedure through pre-loaded images of the instruments/tools required (e.g. dressings, drapes, disposables, instruments such as forceps, retractors, scissors, needle holders, etc)?  (3) Clear explanation through images of the instruments/tools required for each procedure (2) Unclear/confusing images of the instruments/tools at times (1) Completely unclear images of the instruments/tools (0) No pre-loaded images |
| 28 | Does the app contain **correct/accurate** visual explanations for each surgical procedure through pre-loaded images of the instruments/tools (e.g. dressings, drapes, disposables, instruments such as forceps, retractors, scissors, needle holders, etc)?  (3) Accurate explanations through images of the instruments required for each procedure (2) Images of the instruments/tools are not accurate at times (1) Images of the instruments/tools are mostly inaccurate (0) Images of the instruments/tools are totally inaccurate/No pre-loaded images |
| 29 | Does the app contain accurate visual explanation of concepts through pre-loaded **videos** for users to learn (e.g. surgical preparatory procedural videos, surgical steps, instruments, correct usage of the equipment, etc)?  (3) Accurate explanations of concepts through videos (2) Explanations of concepts through videos are not accurate at times (1) Explanations of concepts through videos are mostly inaccurate (0) Explanations of concepts through videos are totally inaccurate/No pre-loaded videos |
| ***C) Credibility: Max score (8 points)*** | |
| 30 | Does the app contain the developer contact details (e.g. email address, contact number, link to website, contact forms)?  (2) App contains more than one contact method for the developer (1) App contains only one contact method for the developer  (0) Contact details are absent |
| 31 | Are the credentials of the developer stated clearly in the app and on the developer’s website (e.g. individual/institution/organisation/university, company/commercial body, etc)?  (2) Credentials are clearly stated on both app and website (1) Credentials are stated either in the app or website (0) Credentials are not stated at all |
| 32 | Does the app contain an accurate description of the contents and functions?  (2) Accurate description of all the contents and functions (1) Only describes some contents/functions or some descriptions are inaccurate  (0) No description of contents and functions or descriptions are mostly inaccurate |
| 33 | Are the sources of funding clearly stated?  (2) All sources of funding are clearly stated  (1) A statement of funding is available, but there is no information about the sources (0) No information about funding |
| ***SECTION 5: PRIVACY AND SECURITY = /4 POINTS*** | |
| 1. ***Security: Max score (2 point)*** | |
| 34 | Does the app require users to register a login profile for authentication purposes (i.e. username and password)?  (2) Requires user login profile with easy registration (1) Requires user login but registration is complex/difficult  (0) Does not require user login profile/registration |
| 1. ***Privacy: Max score (2 points)*** | |
| 35 | Does the app mention clear privacy policies about the collection of data and how it is being used (e.g. whether data is being shared with third parties/advertisers)?  (2) App mentions both collection of users’ data and how it is being used (1) App mentions only collection of users’ data (0) No privacy policy is mentioned |
